# Microbiome and asthma

**DOI:** 10.1186/s40733-017-0037-y

**Published:** 2018-01-05

**Authors:** Milena Sokolowska, Remo Frei, Nonhlanhla Lunjani, Cezmi A. Akdis, Liam O’Mahony

**Affiliations:** 1 Swiss Institute of Allergy and Asthma Research, University of Zürich, Obere Strasse 22, 7270 Davos, Switzerland; 2 Christine Kühne – Center for Allergy Research and Education (CK-CARE), Davos, Switzerland; 3 University of Cape Town, Cape Town, South Africa

**Keywords:** Asthma, Microbiome, Bacteria, Mucosal immune system, Immune tolerance, Short-chain fatty acids, Histamine

## Abstract

The mucosal immune system is in constant communication with the vast diversity of microbes present on body surfaces. The discovery of novel molecular mechanisms, which mediate host-microbe communication, have highlighted the important roles played by microbes in influencing mucosal immune responses. Dendritic cells, epithelial cells, ILCs, T regulatory cells, effector lymphocytes, NKT cells and B cells can all be influenced by the microbiome. Many of the mechanisms being described are bacterial strain- or metabolite-specific. Microbial dysbiosis in the gut and the lung is increasingly being associated with the incidence and severity of asthma. More accurate endotyping of patients with asthma may be assisted by further analysis of the composition and metabolic activity of an individual’s microbiome. In addition, the efficacy of specific therapeutics may be influenced by the microbiome and novel bacterial-based therapeutics should be considered in future clinical studies.

## Background

An enormous number of microbes colonize the skin and mucosal body surfaces. These microbes are highly adapted to survive within complex community structures, utilizing nutrients from other microbes and/or host processes. The microbiome is defined as the sum of these microbes, their genomic elements and interactions in a given ecological niche. The composition and diversity of the microbiome varies across body sites, resulting in a series of unique habitats within and between individuals that can change substantially over time [1]. The establishment of stable microbial communities closely tracks host growth and immune development during the first few years of life. Factors that influence this evolution include antibiotic use, birth mode, infant nutrition and biodiversity in the home, surrounding environment and in family members [2]. Delayed or altered establishment of these microbial communities’ leads to microbiome immaturity and has been associated with increased risk of allergies and asthma later in life.

Highly sophisticated mucosal immune cellular and molecular networks need to be constantly coordinated in order to tolerate the presence of a large number and diversity of bacteria, while protective immune responses to potential pathogens must be maintained and induced on demand. The balance between immune tolerance and inflammation within tissues is regulated in part by the crosstalk between immune cells and the microbiome [3]. Disrupted communication between the microbiome and the host due to altered microbiome composition and/or metabolism is thought to negatively influence immune homeostatic networks. This can be clearly seen in mice bred under germ-free (GF) or sterile conditions, whereby mucosal tolerance mechanisms do not fully develop and these mice display increased allergic responses to allergen challenge.

In this review, we will examine the potential mechanisms by which the microbiome influences immune responses within the lung and assess the evidence for a dysbiotic microbiome in the gut and the respiratory tract of asthma patients. In addition, we will summarize the current therapeutic approaches and challenges associated with microbial-based therapies in asthma patients and highlight the future research and clinical needs in the field.

### Immune mechanisms influenced by the microbiome

Multiple mechanisms have now been described, through which bacteria can induce regulatory responses or dampen inflammatory processes. Both bacterial cell wall components and metabolites from the microbiome have been associated with immunoregulatory effects within the mucosa. Certain commensal microbes such as specific Bifidobacterium, Lactobacillus and Clostridium strains have been shown to increase the proportion of T regulatory cells in mice [4–8]. In addition, Clostridia have been shown to stimulate ILC3s to produce IL-22, which helps to reinforce the epithelial barrier and reduces the permeability of the intestine to dietary proteins [9]. Furthermore, Bifidobacteria and Lactobacilli can stimulate metabolic processes in dendritic cells, such as vitamin A metabolism, tryptophan metabolism and heme oxygenase-1, which promote induction of T regulatory cells [10–12]. The capsular polysaccharide A from *Bacteroides fragilis* has been shown to interact directly with mouse plasmacytoid dendritic cells and thereby promoted IL-10 secretion from CD4+ T cells [13]. In addition, an exopolysaccharide from *Bifidobacterium longum* was recently shown to suppress Th17 responses within the gut and within the lung [14, 15]. Notably, consumption of *Bifidobacterium longum* 35,624 by healthy human volunteers increased Foxp3+ T regulatory cells in peripheral blood, while administration of this bacterial strain to psoriasis patients, chronic fatigue syndrome patients or ulcerative colitis patients consistently resulted in reduced levels of serum proinflammatory biomarkers such as CRP, possibly mediated by increased numbers of T regulatory cells [12, 16].

In addition to bacterial-associated components, bacterial-derived metabolites have significant effects on immunoregulatory processes. Short-chain fatty acids (SCFAs), such as acetate, propionate and butyrate, are produced by the gut microbiota and have been shown to influence dendritic cell and T cell responses, via their binding to G protein-coupled receptors and their inhibition of histone deacetylases, thereby promoting epigenetic changes [17]. Bacteria within the human gut can produce a wide range of biogenic amines (due to metabolism of amino acids), which can also influence immune and inflammatory responses [18]. Interestingly, in murine models, microbiota-derived taurine, histamine, and spermine were shown to influence host-microbiome interactions by co-modulating NLRP6 inflammasome signaling, epithelial IL-18 secretion, and downstream anti-microbial peptide secretion [19].

### Microbiome in animal models of asthma

A number of different animal studies support the concept for a role of the microbiome in development of airway diseases. In particular, valuable insights for the mechanistic role of the microbiome in the development of allergic airway inflammation comes from GF animals, lacking any exposure to pathogenic or nonpathogenic microorganisms. Herbst et al. observed that OVA-induced type 2 airway inflammation and airway hypersensitivity is much stronger in GF mice as compared to the mice from a specific pathogen-free environment (SPF) that were colonized with commensal microbes. Moreover, the exaggerated allergic inflammation in GF mice could be reduced to the same level observed in SPF mice, when GF mice were co-housed for 3 weeks with SPF mice, suggesting that gut and airways recolonization with commensal microbes had protective effects [20]. In addition, early life colonization of GF mice prevented invariant natural killer T cell accumulation in the gut lamina propria and the lungs thereby reducing the severity of allergic airway responses. Later life colonization had no effect on disease phenotypes nor on the development of regulatory T cells or on invariant natural killer T cells [21]. Furthermore, antibiotic treatment of neonatal mice resulted in fewer regulatory T cells and a more pronounced T helper cell type 2 response, which was prevented by re-introducing a commensal intestinal microbiota [22–24].

Gollwitzer et al. examined the susceptibility to house dust mite (HDM)-induced allergic airway inflammation in mice of different ages (3, 15 and 60 days), simulating the conditions of gradual colonisation of the human infant airways [25]. Neonatal mice were prone to develop exaggerated airway eosinophilia, they released more type 2 cytokines and exhibited higher airway hyper-responsiveness following exposure to HDM compared to mice that were older. This protective effect in older mice was associated with the colonization of the mouse lungs with increased numbers of bacteria and the shift from a predominance of Gammaproteobacteria and Firmicutes to Bacteroidetes. The maturation of the lung microbiota was associated with the PDL-1-dependent emergence of Helios-negative T regulatory cells. This study suggests that the absence of specific bacterial species early in life could influence appropriate regulatory mechanisms later in life and subsequently shift the immunological balance towards allergy instead of tolerance [25].

Oral supplementation of mice with specific microbes such as *Bifidobacterium breve*, Clostridium clade IV and XIV species, or with the capsular polysaccharide PSA of *Bacteroides fragilis* induced an anti-inflammatory response associated with induction of regulatory T cells and IL-10 secretion that attenuated allergic airway inflammation [21, 26]. In addition to the gut-induced regulatory T cells that could migrate to the lungs to provide anti-inflammatory effects, there are metabolites produced by the microbiome such as SCFAs that are absorbed and potentially have direct effects on lung immune responses [27]. Deliberate administration of SCFAs, or dietary fibers that are metabolized to SCFAs, has repeatedly been shown to reduce airway inflammation in murine models. A high-fiber diet increased the level of colonic Bacteroidetes and Actinobacteria species and decreased Firmicutes and Proteobacteria, which was associated with increased SCFA serum levels and suppression of allergic airway-inflammation in mice [28]. The beneficial effect was transferred to the offspring after treatment of pregnant mice via epigenetic mechanisms [26, 29]. The influence of the microbiota on D-tryptophan, Vitamin A or biogenic amine metabolism can also modulate T helper cell type 2 mediated allergic airway inflammation within the lung [3, 26, 30, 31].

Several studies have suggested that direct exposure of the murine respiratory tract to microbial products such as endotoxin, CpG-containing oligonucleotides or other Toll-like receptor ligands could inhibit the classical features of asthma [32, 33]. For example, intranasal exposure to the bacterium *Escherichia coli* was protective in the OVA-induced allergic airway inflammation model [34]. These studies were recently expanded by novel findings that linked the protective effect of the farm environment with the microbiota and endotoxin levels in the house dust. Schuijs et al. demonstrated that prolonged exposure to low-dose endotoxin or farm dust protected mice from HDM-induced asthma via A20 (TNFAIP3)-dependent airway epithelial cells-dendritic cells interactions [35]. Stein et al. further demonstrated that intranasal installation of the dust from Amish houses, but not dust from Hutterite homes, reduced OVA-induced allergic airway inflammation in mice, via Myd88 and Trif-dependent mechanisms [36]. The dust from the Amish homes had different bacterial populations (especially higher in Bartonellaceae) and higher endotoxin levels as compared to Hutterite houses’ dust [36].

### The role of the gut microbiome in asthma

The human gut microbiome is the largest collection of bacteria in the body, consisting of 500–1000 distinct bacterial species with more than 8 million genes potentially influencing the host immune system [21, 37]. European adults’ gut microbiota is predominantly colonized by Bacteroidetes, Firmicutes, Actinobacteria, Proteobacteria, and Verrucomicrobia. The stomach, duodenum, and proximal small intestine are mainly colonized with aerobic bacteria including Streptococci species, Lactobacilli species, and Enterobacteriaceae while anaerobes such as Bacteroides, Bifidobacterium, Prevotellaceae, Rikenellaceae, Lachnospiraceae, Ruminococcaceae, and Clostridium species dominate the distal small intestine and the colon [26, 38]. The gut microbiota can influence immune responses at distant sites (such as the lung) via multiple mechanisms. For example, it was recently shown that there is an increase in the number of bacteria capable of secreting histamine from the gut of adult asthma patients, compared to healthy volunteers [39]. However, it is not clear if increased secretion of histamine by gut microbes can have an overall detrimental or protective effect as histamine can induce protective responses in the lung via histamine 2 receptor and detrimental effects via histamine 1 and 4 receptors [40].

The composition of the gut microbiome is thought to reach an adult-like diversity by 3 years of age. Development of the early life gut microbiome is influenced by many environmental factors, such as living in microbial rich environments (e.g. on a farm or with frequent contact to livestock and pets), or a diverse diet, which have been inversely associated with childhood asthma [41–45]. It is thought that exposure to and colonization by certain microbes at the correct time during early life is important for gut development, immune cell maturation and resistance to pathogens, all of which may protect against the development of asthma [22, 37, 46]. The mode of delivery has a significant influence on colonization. Babies delivered via caesarean section typically have more Staphylococcus species, Bacillales, Propionobacterineae, Corynebacterineae, Firmicutes and Acinetobacter species with fewer Actinobacteria and Bacteroidetes, while vaginal delivery has been linked to increased colonization with Clostridia [38, 47]. Clostridia metabolize fibers to SCFAs, which can have systemic anti-inflammatory effects as described above. In addition to delivery mode and diet, maternal antibiotic use during pregnancy or antibiotic treatment in early childhood significantly disrupts the microbiota and was associated with long-lasting effects such as decreased Actinobacteria and increased Bacteroidetes and Proteobacteria [1].

Several studies have linked early life dysbiosis of the gut microbiota with an altered risk of asthma later in life. Colonization by *Clostridium difficile* at 1 month of age was associated with wheeze throughout the first 6 to 7 years of life and with asthma at age 6 to 7 years [48]. Children that developed asthma at school age, had a lower gut microbiome diversity at 1 week or 1 month of age, but not at 1 year of age, compared to non-asthmatic children [49]. In another study, the early life relative abundance of the bacterial genera Lachnospira, Veillonella, Faecalibacterium, and Rothia was significantly decreased in children at risk of asthma. This dysbiosis was accompanied by reduced levels of fecal acetate and dysregulation of enterohepatic metabolites [50]. In addition, neonates with the lowest relative abundance of Bifidobacteria, Akkermansia and Faecalibacterium and a higher relative abundance of particular fungi (Candida and Rhodotorula), had the highest risk to develop atopy and asthma [51]. Thus, early life dysbiosis of the gut microbiota has been consistently associated with an increased risk of asthma later in life. However, it remains unclear if microbial dysbiosis within the gut can actually drive relevant disease promoting mechanisms or if dysbiosis simply reflects associated phenomena such as altered patterns of immune response to microbes and environmental stimuli.

### Role of the respiratory microbiome in asthma

The Human Microbiome Project, launched in 2007, did not include airway tissue sampling as healthy human lung tissue at that time was assumed to be sterile [52]. However, shortly afterwards a number of pioneering publications in this field appeared and several research consortia and individual groups subsequently started intensive studies to characterize and understand the composition and function of airway microbiota in health and disease [53–55]. Currently, it is known that the healthy respiratory mucosa is inhabited by niche-specific bacterial communities [56]. The highest densities of bacterial communities are found in the upper respiratory tract, reaching up to 10^3^ viable bacteria per nasal swab from the nasal cavity and nasopharynx, with even up to 10^6^/ml viable cells from oropharynx lavages [56–58]. In the trachea and lungs, the estimated numbers of bacteria are lower with approximately 10^2^ bacterial cells per ml being found in bronchoalveolar lavages (BAL) from healthy lungs [59]. The six dominant phyla routinely found in the lung are Firmicutes, Proteobacteria, Bacteroides, Fusobacteria, Acidobacteria, and Actinobacteria [60].

The original proof-of-concept study from Hilty et al., with microbiome assessments of the nose, oropharynx, bronchial brushings and BAL samples from the lower airways revealed that the Proteobacteria phylum and especially Haemophilus species are more often present in upper and lower airways of asthmatic and COPD adults and asthmatic children [53]. The study performed by Huang et al. in patients with suboptimal controlled asthma, defined as persistent symptoms on the Asthma Control Questionnaire after 4 weeks of standardized treatment with inhaled fluticasone, showed a greater airway microbiota diversity in these patients compared to control subjects that correlated positively with bronchial hyperresponsiveness [61]. Specifically, there was an increase in the phylum Proteobacteria in asthma patients, including Comamonadaceae, Sphingomonadaceae, Nitrosomonadaceae, Oxalobacteraceae, and Pseudomonadaceae families [61]. Interestingly, adult patients who benefited most from clarithromycin treatment, as assessed by the reduction in bronchial hyperactivity to methacholine were those who had significantly greater bacterial diversity prior the intervention [61]. Subsequent studies also confirmed that Proteobacteria were present in higher proportions in the asthmatic airways [59, 62]. In addition, Klebsiella species were enriched in patients with severe asthma as compared to patients with mild-to-moderate asthma and controls [63]. Moreover, within severe asthma patients, Proteobacteria was associated with T_H_17-related gene signature in airway epithelium, worsening asthma control and total leucocytes in the sputum, while Bacteroides/Firmicutes were more abundant in obese patients with severe asthma. In contrast, the presence of Actinobacteria correlated with improvement and/or no change in asthma control [63]. Severe asthma had long been associated with the presence of *Mycoplasma pneumoniae* and *Chlamydophila pneumoniae*, resulting in several clinical trials with macrolide antibiotics in this group of patients [64]. Yet, in the face of controversial study results and the possibility that beneficial microbial species are also affected, further clinical trials that include detailed microbiome studies are needed [65, 66].

The composition of the airway microbiome develops exponentially very early in life and later in life can be influenced by the environment, health status and age. Birth mode (vaginal or via caesarean section), the exposures during the first hours of life and the environment of the following 3–4 months of life have been shown to be of utmost importance in shaping the development of stable respiratory and gut microbiota to ensure respiratory health later in life [50, 67–69]. Both human and animal studies have shown that inhaled dust particles can carry a complex mixture of microbes and microbial factors, which influence susceptibility to asthma development via their effects on innate and adaptive immune responses [35, 36]. The important research questions that are currently being addressed in children include: i) what is the longitudinal process of upper airways colonization in healthy infants? ii) how do environmental factors such as breast feeding, living on a farm, number of siblings, day-care, pets at home, smoking and antibiotic usage impact the respiratory microbiome? iii) are there correlations between patterns of respiratory microbial colonization in early life with the occurrence of acute respiratory infections such as respiratory syncytial virus (RSV), rhinovirus and influenza virus and their further impact on chronic non infectious-associated recurrent wheeze, atopic sensitization and asthma? [58, 70–74].

Teo et al. analyzed the nasopharynx microbiome in a prospective cohort of children at several time-points up to 2 years of age and correlated the presence of specific groups of bacteria with acute respiratory infections [68]. Healthy infants from this cohort were initially colonized with Staphylococcus or Corynebacterium species up to 2 months of age with subsequent stable colonization by Allociococcus or Moraxella. In contrast, Streptococcus, Moraxella or Haemophilus colonization were correlated with virus-associated acute respiratory infections in the first 60 weeks of life. Early asymptomatic Streptococcus colonization, rare in children from dog and cat-owning families, increased the risk of asthma at 5 years of age [68]. Early upper respiratory tract colonization with *S. pneumoniae*, *H. influenzae* and/or *M. catarrhalis* in children at 4 weeks of age from other prospective birth cohorts was also found to be associated with an increased risk of pneumonia and bronchiolitis or asthma at 5 years of age [54, 75]. Additional studies have also noted associations between *H. influenza*, Streptococcus species and *S. aureus* nasopharyngeal colonization with RSV infection and hospitalization in children independently of their age [76–78]. Furthermore, early colonization of the upper respiratory tract of healthy infants with Staphylococcus species, subsequently followed by Corynbactrium/Dolosigranulum and Moraxella, were described for infants who were breastfed and who had lower rates of respiratory infections in the first 2 years of life [67, 79, 80]. Indeed, airway microbial diversity appears to be inversely associated with sensitization to house dust mites in early childhood [81, 82]. Of particular interest is a recent study comparing Amish children raised on traditional farms, who have a low prevalence of asthma and atopy, with Hutterite children coming from highly industrialized farms who have a higher prevalence of asthma and atopy, even though these two populations are genetically similar. One striking difference was the microbial composition and endotoxin load of dust from those two housing environments, associated with the enhanced induction of innate immune pathways in Amish children. The high-endotoxin dust from Amish houses was able to inhibit OVA-induced allergic airway inflammation in mice, as described above [36]. Several other studies have also confirmed that the farm environment is associated with increased bacterial diversity in the house dust samples and nasal microbiome diversity of the same children who have lower risk of developing asthma [83–85]. It is currently unknown if the protective effect of the dust-associated microbiome is due to inhalation of multiple bacteria species and further colonization of the airways, or if inhaled bacterial metabolites may also play a role.

### Microbiome strategies for asthma prevention, treatment and management

Alterations in the lung and gut microbiome of asthma patients have been well described previously in this review. The deliberate restoration of lung and gut microbiota through the use of prebiotics; probiotics or synbiotics is one potential strategy currently being assessed. Interest in probiotics and prebiotics for their potential benefits in protecting against airway inflammation is relatively recent but increasing significantly, particularly as several lines of evidence suggest that a “healthy” microbial community facilitates the development of immune tolerance [30]. In vitro studies and animal models have repeatedly shown the protective effects of certain probiotic strains on lung inflammatory responses, but have also shown that not all probiotics will induce the same effects [86]. Intervention and prevention studies in humans are inconsistent in their findings, possibly because many factors complicate the analysis of dysbiosis in patients with asthma. Comparison between human studies are difficult, because of considerable heterogeneity in the probiotics and/or prebiotics used, study design, sample size, age of study population, geographic location and lifestyle factors (including diet). One preliminary study did suggest that symbiotic (pre and probiotic) use improved peak expiratory flow and reduced the systemic production of Th2 cytokines in allergic asthmatics [87]. Another recent study using a combination of nutritional interventions (fish oils and vegetable extracts) with a probiotic led to significant improvement in pulmonary function parameters and significantly reduced requirement for short-acting inhaled bronchodilators and inhaled corticosteroids in children with asthma, suggesting that the combination of multiple approaches may lead to the most optimal benefits [88]. These findings are promising, however more definitive studies are needed to determine whether modification of gut and lung microbiota can be attributed to pre and/or probiotic use. Currently, there is no recommendation to use pre- or probiotics for treatment or prevention of asthma. Nevertheless, there is accumulating evidence that antenatal interactions between maternal diet, gut bacteria and bacterial metabolites may lead to immunological imprinting on the developing fetal immune system that could influence the development of allergy and asthma later in life [89]. Thus, further studies are required to determine if appropriate prebiotic and probiotic use during pregnancy may functionally impact the maternal gut microbiome with subsequent effects on maternal immune function and risk of asthma in the offspring [90].

In addition to using single probiotic bacterial strains, the manipulation of the entire gut microbiome with fecal microbiota transplants (FMT) is currently being explored. FMT has been successfully used for the treatment of *Clostridium difficile* infection and research into its use for other inflammatory diseases such as type 2 diabetes, inflammatory bowel diseases and non-alcoholic steatohepatitis is well under way [91]. The use of FMT beyond intestinal disorders requires additional studies and currently there is no data supporting its use in allergic disease or asthma [1].

The role of the microbiome in influencing precision medicine approaches to patient care has been best explored to date in the oncology field. Accumulating evidence suggests that the microbiome not only influences the severity of treatment-associated side effects in cancer patients, but also has a dramatic effect on treatment efficacy via pharmacodynamic and immunological mechanisms [92]. Notably, a melanoma mouse model showed commensal microbe-derived antitumor immunity evidenced by higher intratumoral CD8+ T cell accumulation. From this microbiota, Bifidobacteria were identified as having the strongest association with antitumor T cell immunity and the ability to maximize the efficacy of the cancer immunotherapeutic anti-PD-L1-specific antibody treatment. Bifidobacteria augmented dendritic cell function leading to enhanced CD8+ T cell priming and accumulation within the tumor [93]. While there is a growing amount of data on the compositional differences in lung microbiota in health and disease, there is a dearth of research into the functional role of the microbiome on treatment efficacy in patients with chronic respiratory disorders [94]. One important study did correlate corticosteroid use and corticosteroid sensitivity in asthma patients with the presence of specific microbes in the lower airways. At the genus level, Neisseria species, Haemophilus species, Campylobacter species and Leptotrichia species were present in the lower airways of patients with corticosteroid-resistant asthma, but not in patients with corticosteroid-sensitive asthma [59]. Others have demonstrated that corticosteroid use, particularly the combination of inhaled and oral corticosteroids, is associated with an increased abundance of Proteobacteria and the genus Pseudomonas, and decreased abundance of Bacteroidetes, Fusobacteria, and Prevotella species [60]. One recent study suggests that microbiome-related functions might affect responsiveness to corticosteroid treatment in asthma patients [95]. Pre-steroid treatment Haemophilus levels were increased in asthma patients with diminished responses to corticosteroids. Furthermore, the predicted metagenome metabolic functions in inhaled corticosteroid nonresponders suggested increased microbiome-associated xenobiotic degradation capacity [95].

Further profiling and characterization of the microbiome associated with different asthma phenotypes is necessary for identifying novel microbiota-related mechanisms of disease. In addition, identification of these key microbial species and their associated functional effects will contribute to a more precise definition of asthma phenotypes and may help identify more suitable “phenotype-specific” management strategies [96].

### Future perspectives

While it is clear that the microbiome significantly influences host immune maturation and immune activity, the molecular basis for these immunomodulatory mechanisms are only beginning to be elucidated. It still remains unclear whether and, if so, to what extent patterns of airway microbial dysbiosis actually drives rather than merely reflects associated patterns of immune reactivity within the lung. Current studies in prospective birth cohorts and cross-sectional studies in children have heightened our awareness of time-sensitive patterns of colonization of seemingly protective or detrimental bacteria in the gut or airways of healthy and diseased children. However, further mechanistic and epidemiological studies are needed to uncover the functional, multidirectional associations between the specific bacterial strains, host, allergens and viruses. Respiratory microbiome assessments in adults have so far been performed in a cross-sectional manner, comparing the airway microbiota composition between healthy controls and patients with asthma and often with other chronic airway diseases. Some studies have provided detailed clinical characteristics of patients, allowing for the correlation of microbiota differences across different asthma phenotypes. However, longitudinal and prospective analyses of adult airway microbiome in bigger cohorts of well-characterized patients are still needed to understand the relationships between the course of the disease, its phenotype and endotype, susceptibility to exacerbations and disease progression as well as its response to treatment.

Compositional profiling needs to be complemented with metagenomics, transcriptomics, physiological, biochemical and function-oriented analyses of both the host response and microbial communities as interactions between the host and microbiome are almost certainly bidirectional, with species- and strain-specific behaviors shaped by the genetic background and microenvironment in which they exist. In addition, current compositional analysis at the genus level is not sufficient and future analysis needs to be conducted at greater depth to include information at the species and strain level. The immune response to a bacterium is often strain-specific and results from one strain cannot be extrapolated to other strains even within the same species. Thus, the traditional methods for microbiological classification, based on 16 s sequencing and certain biochemical properties, of a bacterium into a given genus or species do not currently help us to predict immunological outcomes. Culturing methods need also to be improved in order to isolate and grow lung-derived bacterial strains in vitro, particularly the obligate anaerobes, in order to facilitate strain-specific immunological assessments.

## Conclusions

The last few years have resulted in pivotal studies that clearly associate changes in gut or lung microbial populations with asthma. However, mechanistic studies are still necessary to elucidate how members of the microbiota induce or modulate inflammatory responses in asthmatic patients. We anticipate that the continuing identification of novel bacterial strains, their components and metabolites, which modulate mucosal immunoregulatory responses, will open up new possibilities for a bug-to-drug approach in the treatment of asthma patients and the prevention of airway diseases.

## Abbreviations

BAL: Bronchoalveolar lavages
FMT: Fecal microbiota transplant
GF: Germ-free
HDM: House dust mite
NLRP6: NOD-like receptor family pyrin domain containing 6
RSV: Respiratory syncytial virus
SCFAs: Short-chain fatty acids
SPF: Specific pathogen-free
